# *N*‐acetyltransferase 10 facilitates tumorigenesis of diffuse large B‐cell lymphoma by regulating AMPK/mTOR signalling through N4‐acetylcytidine modification of SLC30A9

**DOI:** 10.1002/ctm2.1747

**Published:** 2024-07-03

**Authors:** Mengfei Ding, Zhuoya Yu, Tiange Lu, Shunfeng Hu, Xiangxiang Zhou, Xin Wang

**Affiliations:** ^1^ Department of Hematology, Shandong Provincial Hospital Shandong University Jinan Shandong China; ^2^ Department of Hematology Shandong Provincial Hospital, Affiliated to Shandong First Medical University Jinan Shandong China; ^3^ National Clinical Research Center for Hematologic Diseases the First Affiliated Hospital of Soochow University Suzhou China; ^4^ Taishan Scholars Program of Shandong Province Jinan Shandong China; ^5^ Branch of National Clinical Research Center for Hematologic Diseases Jinan Shandong China

**Keywords:** Diffuse large B‐cell lymphoma, N4‐acetyclytidine, *N*‐acetyltransferase 10, Solute carrier family 30 member 9

## Abstract

**Background:**

Accumulating studies suggested that posttranscriptional modifications exert a vital role in the tumorigenesis of diffuse large B‐cell lymphoma (DLBCL). N4‐acetylcytidine (ac4C) modification, catalyzed by the *N*‐acetyltransferase 10 (NAT10), was a novel type of chemical modification that improves translation efficiency and mRNA stability.

**Methods:**

GEO databases and clinical samples were used to explore the expression and clinical value of NAT10 in DLBCL. CRISPER/Cas9‐mediated knockout of NAT10 was performed to determine the biological functions of NAT10 in DLBCL. RNA sequencing, acetylated RNA immunoprecipitation sequencing (acRIP‐seq), LC‐MS/MS, RNA immunoprecipitation (RIP)‐qPCR and RNA stability assays were performed to explore the mechanism by which NAT10 contributed to DLBCL progression.

**Results:**

Here, we demonstrated that NAT10‐mediated ac4C modification regulated the occurrence and progression of DLBCL. Dysregulated *N*‐acetyltransferases expression was found in DLBCL samples. High expression of NAT10 was associated with poor prognosis of DLBCL patients. Deletion of NAT10 expression inhibited cell proliferation and induced G0/G1 phase arrest. Furthermore, knockout of NAT10 increased the sensitivity of DLBCL cells to ibrutinib. AcRIP‐seq identified solute carrier family 30 member 9 (SLC30A9) as a downstream target of NAT10 in DLBCL. NAT10 regulated the mRNA stability of SLC30A9 in an ac4C‐dependent manner. Genetic silencing of SLC30A9 suppressed DLBCL cell growth via regulating the activation of AMP‐activated protein kinase (AMPK) pathway.

**Conclusion:**

Collectively, these findings highlighted the essential role of ac4C RNA modification mediated by NAT10 in DLBCL, and provided insights into novel epigenetic‐based therapeutic strategies.

## Graphical abstract

- NAT10 expression is elevated in DLBCL, and its high expression is associated with poor prognosis.
- NAT10 exerts tumour‐promoting effects by regulating cell proliferation and cell cycle.
- NAT10 promotes tumorigenesis of DLBCL by regulating AMPK/mTOR signalling through ac4C modification of SLC30A9.

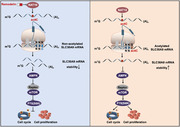

## 1 INTRODUCTION

Diffuse large B‐cell lymphoma (DLBCL) is a type of lymphoma with highly heterogeneous phenotype and molecular characteristics.^1^ It is also the most common type of aggressive non‐Hodgkin's lymphoma (NHL).^2^ Despite the good therapeutic effect of standard R‐CHOP therapeutic regimen for DLBCL, about 30% of the patients suffer from relapse or refractory disease.^3^, ^4^, ^5^, ^6^ Both genetic and epigenetic aberrations contribute to the progression of DLBCL.^7^ Accumulating studies suggest that targeting epigenetic heterogeneity offers the possibility of broadly modulating transcriptional activity of tumour cells and may exert a promising therapeutic strategy in DLBCL.^8^

RNA modifications play crucial roles in regulating mRNA stability and translation efficiency in cancer cells.^9^ N4‐acetylcytidine (ac4C) is a novel modification with highly conserved that exists in a variety of RNAs.^10^, ^11^ It was initially discovered in the bacterial tRNA^met^ anticodon^12^ and subsequently reported in the mammalian 18S rRNA decoding site and in the eukaryotic D‐stem of tRNA^Ser/Leu^.^13^, ^14^ Recently, it has been reported as a marker of human mRNAs and could enhance the stability and translation efficiency of mRNA.^15^, ^16^ At present, significant alteration in ac4C levels has been reported in quantities of cancers, suggesting that ac4C modification is essential for tumorigenesis.^17^, ^18^, ^19^, ^20^

As a lysine acetyltransferase, *N*‐acetyltransferase 10 (NAT10) belongs to the family of general control non‐repressible 5‐related *N*‐acetyltransferases. It was recently discovered that NAT10 is the only enzyme capable of catalyzing the ac4C modification of RNA.^21^ The RNA acetyltransferase NAT10 has been implicated in multiple diseases through its role as an RNA acetyltransferase.^22^, ^23^, ^24^, ^25^ NAT10 is reported to contribute to several biological processes, such as histone acetylation, DNA damage response, telomerase activity and mitotic cell fate.^20^, ^26^ Solute carrier family 30 member 9 (SLC30A9) is one of the SLC30A (solute carrier 30A) family members. A prior study demonstrated that SLC30A9 interacted with β‐catenin and enhanced its transcriptional activity in the Wnt signalling pathway,^27^ which activation is well known to affect DLBCL development and progression.^5^ However, the dysregulation and underlying mechanism of ac4C modification mediated by NAT10 in DLBCL have not yet been elucidated.

Herein, we investigated the association between NAT10 expression and the clinicopathological characteristics of DLBCL patients, clarified the role of NAT10‐mediated ac4C modification in DLBCL. Mechanistically, NAT10 contributed to the development of DLBCL by regulating AMPK signalling via the ac4C modification of SLC30A9 mRNA. Our findings indicated that NAT10 might serve as a novel therapeutic target for DLBCL progression by revealing the biological role of ac4C modification.

## 2 RESULTS

### 2.1 Elevated expression of NAT10 in DLBCL and association with poor outcome

**FIGURE 1:**
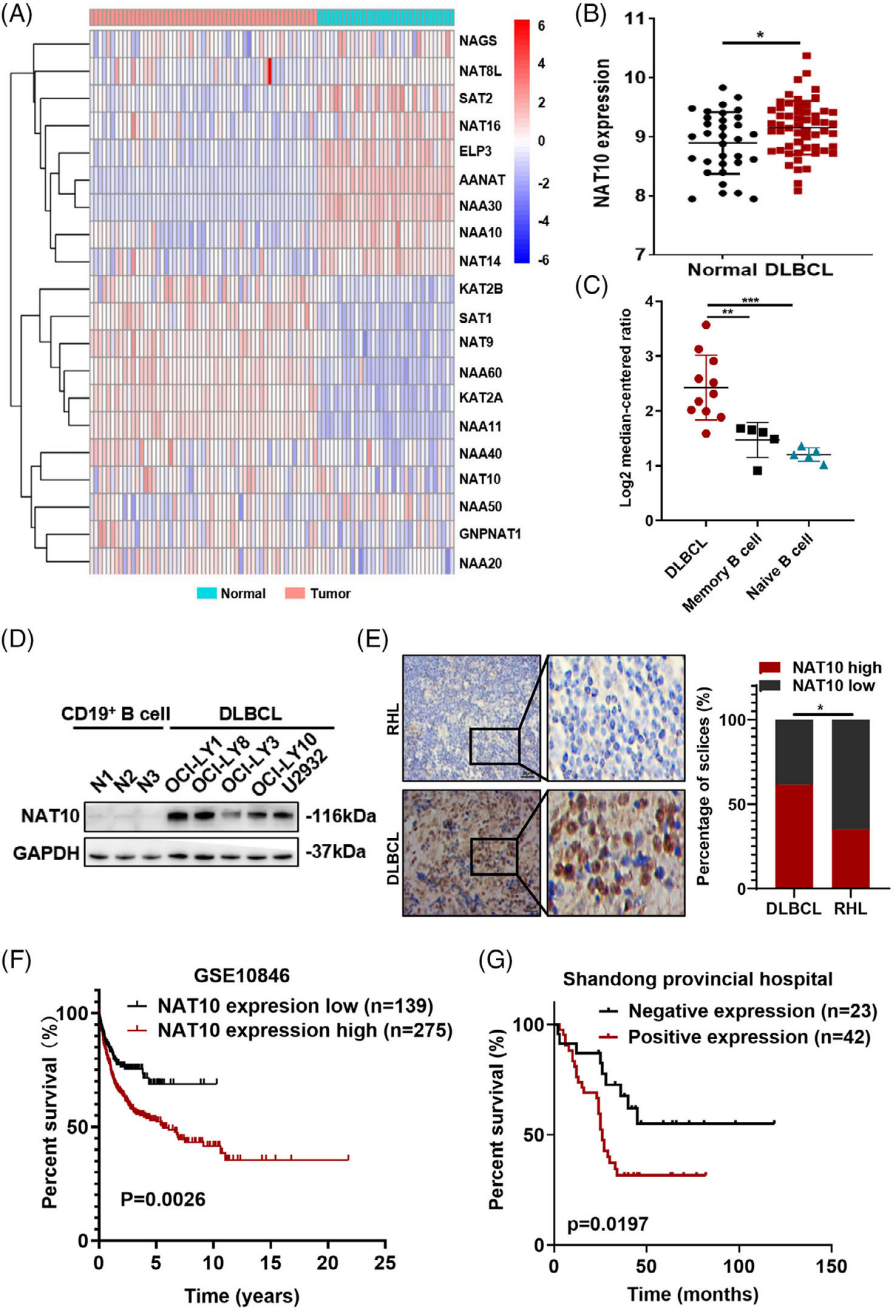
Dysregulated expression of *N*‐acetyltransferases and association with poor prognosis of DLBCL patients. (A) Heatmap of *N*‐acetyltransferases expression in normal (healthy tonsil tissues, *n* = 33) and DLBCL tissues (*n*  =  55) from the GEO database (GSE56315), with high and low expression levels shown in red and blue, respectively. (B) NAT10 was upregulated in DLBCL tissues (*n*  =  55) compared to normal samples (healthy tonsil tissues, *n*  =  33) based on the GSE56315 dataset. *p < .05. *p*‐Values came from unpaired two‐tailed *t*‐test. (C) The expression of NAT10 in the Oncomine dataset. **p < .01, ***p < .001. *p*‐Values came from unpaired two‐tailed *t*‐test. (D) Western blot analysis of the NAT10 protein expression level in different DLBCL cell lines. (E) Representative images of immunohistochemical staining for the NAT10 protein (left). Compared to samples of reactive hyperplasia lymphoid (RHL), expression level of NAT10 was significantly increased in DLBCL tissues. NAT10 is mainly localized in the nucleus. Bar  =  50 µm. Statistical analysis of NAT10‐positive staining in patients with DLBCL (*n* = 104) and RHL (*n* = 20) (right). *p < .05. *p*‐Value came from the chi‐square test. (F) Kaplan–Meier survival curve analysis indicated that high NAT10 expression in DLBCL appeared to be correlated with a shorter overall survival (OS) based on analysis of the GSE10846 dataset (*p*  =  .0026). The median OS was assessed by the log‐rank test. (G) Kaplan–Meier survival curve analysis indicated that high NAT10 expression in DLBCL appeared to be correlated with shorter OS based on analysis of our IHC data (*n*  = 65, *p*  =  .0197). The median OS was assessed by the log‐rank test.

Expression levels of *N*‐acetyltransferases were detected (GSE56315), and the results revealed that several *N*‐acetyltransferases were abnormally expressed in DLBCL patients (Figure 1A). Given that NAT10 is the only confirmed regulator of ac4C modification,^15^ we further investigated its function in DLBCL. A significant increase in NAT10 mRNA levels was detected in GSE56315 (Figure 1B) and confirmed by the Oncomine database (Figure 1C). Compared with normal CD19^+^ B cells, DLBCL cells express a higher level of NAT10 protein (Figure 1D).

We further investigated the relationship between NAT10 protein level and the clinicopathological characteristics of patients with DLBCL. NAT10 protein level was detected by immunohistochemistry (IHC) in primary DLBCL samples from Shandong Provincial Hospital (SPH). Compared with reactive hyperplasia lymphoid (RHL), DLBCL tissues displayed remarkably increased NAT10 expression (64/104 vs. 7/20, *p* = .0280; Figure 1E). Among the included DLBCL patients, 79 patients with complete and available clinical information were selected for further analysis of clinicopathological characteristics, of whom 65 patients were followed up (Table 1). A treatment response analysis of enrolled patients was conducted in order to clarify the impact of NAT10 on clinical management. Of the 104 patients enrolled in the study, 39 completed an outcome assessment, with 76.9% (30/39) of complete remissions (CR) and partial remissions (PR). No significant impact of NAT10 expression on clinical efficacy (Table 1). There was a positive relationship between NAT10 and the international prognostic index (IPI, *p* = .0267) in these patients with complete clinical information. Survival analyses of GSE10846 dataset revealed increased NAT10 expression was associated with shorter overall survival (OS) in DLBCL patients (*p* = .0026, Figure 1F), suggesting the potential prognostic value of NAT10 in DLBCL. Compared to patients without high NAT10 expression, NAT10‐positive patients had notably shorter OS (*p* = .0197, Figure 1G), consistent with the results of GSE10846. These results indicate the dysregulated expression and prognostic value of NAT10 in DLBCL.

**TABLE 1:** The clinical information of DLBCL primary samples of SPH.

		NAT10 expression		
Variables	No. of patients	Positive	Negative	*p*‐Value
Age (years)				
** ≤60**	43	23	20	.3213
** >60**	34	22	12	
Gender				
** Male**	37	19	18	.1935
** Female**	41	27	14	
Ann Arbor stage				
** I or II**	24	13	11	.8333
** III or IV**	44	25	19	
B symptoms				
** Present**	27	17	10	.4478
** Absent**	41	22	19	
Subtype				
** GCB**	20	12	8	.6835
** Non‐GCB**	44	24	20	
Serum LDH				
** Normal**	30	15	15	.2944
** Elevated**	45	28	17	
Extranodal involvement				
** Absent**	22	15	7	.7057
** Present**	55	30	25	
IPI score				
** 0–2**	32	15	17	.0267*
** 3–5**	40	29	11	
Treatment responses				
** CR or PR**	30	18	12	.8121
** PD or SD**	9	5	4	

### 2.2 Inhibition of NAT10 suppressed cellular proliferation of DLBCL both in vitro and in vivo

**FIGURE 2:**
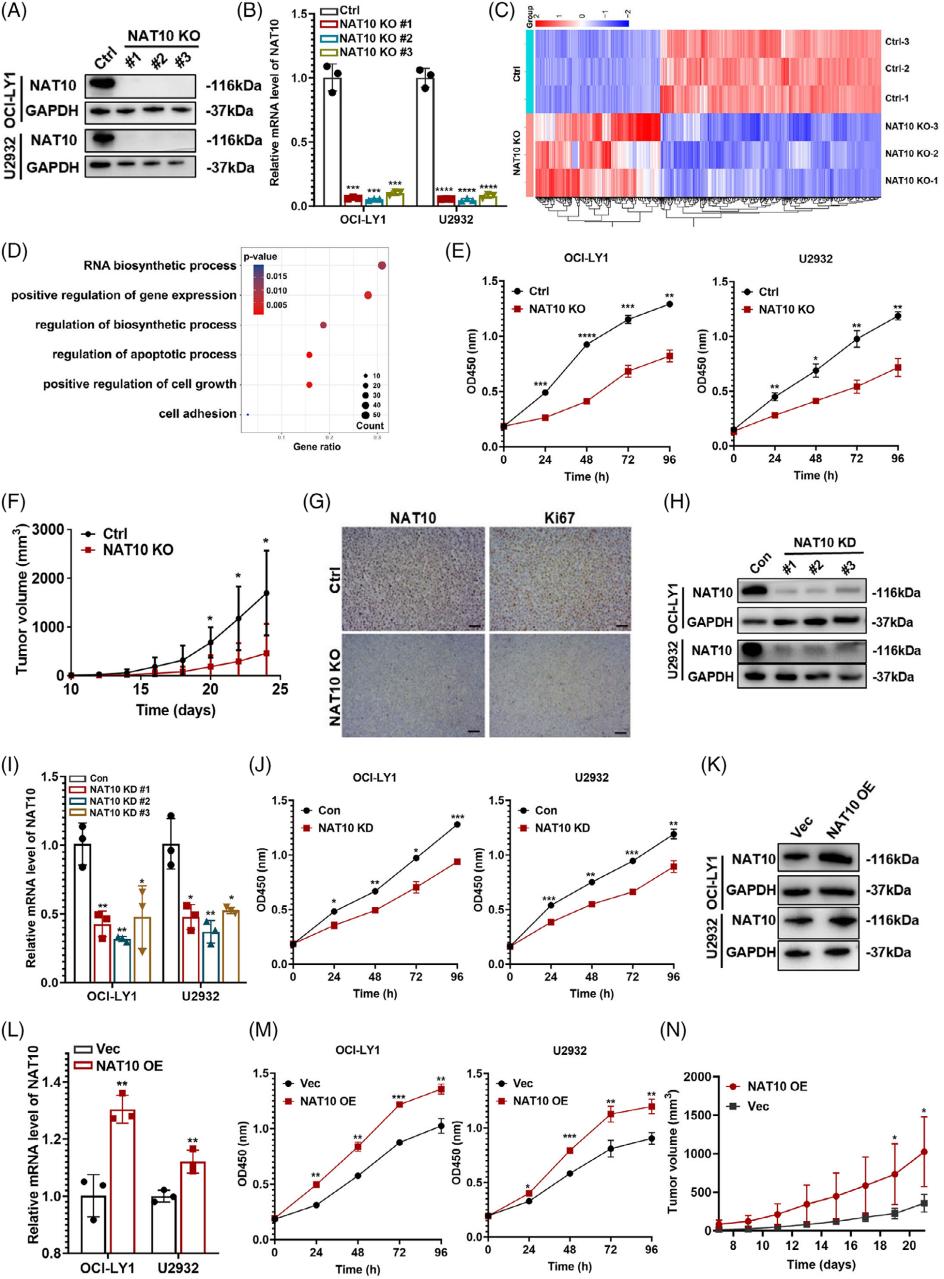
NAT10 promoted cellular proliferation of DLBCL cells both in vitro and in vivo. (A) Western blot analysis confirmed CRISPR/Cas9‐mediated NAT10 deletion in OCI‐LY1 and U2932 cells. Ctrl: negative control (non‐target gRNAs), NAT10 KO: CRISPR/Cas9 gene editing system targeting deletion of NAT10 (NAT10 knockout). (B) qRT‐PCR assay confirmed CRISPR/Cas9‐mediated NAT10 deletion in OCI‐LY1 and U2932 cells. Ctrl: negative control (non‐target gRNAs), NAT10 KO: CRISPR/Cas9 gene editing system targeting deletion of NAT10 (NAT10 knockout). At least three independent experiments were conducted to obtain the data presented as mean  ±  SD. ***p < .001. ****p  <  .0001. *p*‐Values from unpaired two‐tailed *t*‐test. (C) Differentially expressed genes from RNA‐seq of OCI‐LY1 cells with or without NAT10 knockout, (*n* = 3). Ctrl: negative control (non‐target gRNAs), NAT10 KO: CRISPR/Cas9 gene editing system targeting deletion of NAT10 (NAT10 knockout). (D) Functional enrichment analyses of differentially expressed genes according to RNA‐seq of OCI‐LY1 cells with or without NAT10 knockout, *n* = 3. Ctrl: negative control (non‐target gRNAs), NAT10 KO: CRISPR/Cas9 gene editing system targeting deletion of NAT10 (NAT10 knockout). (E) Relative proliferation levels of OCI‐LY1 and U2932 cells transfected with Ctrl or NAT10 KO detected by CCK‐8 assay. Ctrl: negative control (non‐target gRNAs), NAT10 KO: CRISPR/Cas9 gene editing system targeting deletion of NAT10 (NAT10 knockout). At least three independent experiments were conducted to obtain the data presented as mean  ±  SD. *p < .05, **p < .01, ***p < .001. ****p  <  .0001. *p*‐Values from two‐way ANOVA with Sidak correction. (F) SCID beige mice were subcutaneously injected with OCI‐LY1 cells with or without NAT10 knockout (*n*  =  6). Ctrl: negative control (non‐target gRNAs), NAT10 KO: CRISPR/Cas9 gene editing system targeting deletion of NAT10 (NAT10 knockout). *p < .05. *p*‐Values from two‐way ANOVA with Sidak correction. (G) IHC staining with NAT10 and Ki67 expression levels were performed from NAT10 knockout xenograft tumour tissues (*n* = 3). Bar  =  50 µm. Ctrl: negative control (non‐target gRNAs), NAT10 KO: CRISPR/Cas9 gene editing system targeting deletion of NAT10 (NAT10 knockout). (H) OCI‐LY1 and U2932 cells were stably transfected with NAT10 knockdown lentivirus (shNAT10, NAT10 KD) and control (Con). The lentivirus‐mediated NAT10 repression was confirmed by Western blotting. (I) OCI‐LY1 and U2932 cells were stably transfected with NAT10 knockdown lentivirus (shNAT10, NAT10 KD) and control (Con). The lentivirus‐mediated NAT10 repression was confirmed by qRT‐PCR. At least three independent experiments were conducted to obtain the data presented as mean  ±  SD. ***p < .001. ****p <  .0001. *p*‐Values from unpaired two‐tailed *t*‐test. (J) NAT10 knockdown decreased cellular proliferative activity in DLBCL. Con: control, NAT10 KD: lentivirus‐mediated NAT10 knockdown. At least three independent experiments were conducted to obtain the data presented as mean  ±  SD. *p < .05, **p < .01, ***p < .001. *p*‐Values from two‐way ANOVA with Sidak correction. (K) OCI‐LY1 and U2932 cells were stably transfected with NAT10 overexpression lentivirus (NAT10 OE) and empty vector (Vec). The overexpression efficiency of NAT10 was confirmed by western blot. (L) OCI‐LY1 and U2932 cells were stably transfected with NAT10 overexpression lentivirus (NAT10 OE) and empty vector (Vec). The overexpression efficiency of NAT10 was confirmed by qRT‐PCR. At least three independent experiments were conducted to obtain the data presented as mean  ±  SD. **p < .01. *p*‐Values from unpaired two‐tailed *t*‐test. (M) NAT10 overexpression increased cellular proliferative activity in DLBCL. Vec: empty vector, NAT10 OE: lentivirus‐mediated NAT10 overexpression. At least three independent experiments were conducted to obtain the data presented as mean  ±  SD. *p < .05, **p < .01, ***p < .001. *p*‐Values from two‐way ANOVA with Sidak correction. (N) SCID beige mice were subcutaneously injected with OCI‐LY1 cells with or without NAT10 overexpression (*n*  =  5). Vec: empty vector, NAT10 OE: lentivirus‐mediated NAT10 overexpression. *p < .05. *p*‐Values from two‐way ANOVA with Sidak correction.

We further evaluated biological function of NAT10 in DLBCL cells. CRISPR/Cas9 genome editing system was employed to delete NAT10 in DLBCL cells, of which sgNAT10#2 (NAT10 knockout, NAT10 KO) exhibited the highest efficacy (Figure 2A,B) and was selected for subsequent functional experiments. RNA‐seq was performed to further clarify NAT10‐related biological processes in OCI‐LY1 DLBCL cell lines. The RNA‐seq screened 896 differentially expressed genes (DEGs) in OCI‐LY1 cells with NAT10 knockout, of which 324 were downregulated and 572 were upregulated, respectively (Figure 2C). GO enrichment analysis uncovered that NAT10 was involved in cell apoptosis, RNA biosynthesis and cell growth (Figure 2D). DLBCL cells with NAT10 knockout demonstrated significant inhibition of cell proliferation (Figure 2E). To investigate its role in tumorigenicity in vivo, OCI‐LY1 cells with negative control (Ctrl) and NAT10 knockout (NAT10 KO) were used to create xenograft mouse models. NAT10 knockout significantly decreased tumour growth (Figure 2F). IHC assay also revealed the reduced expression of NAT10 as well as decreased proliferation‐associated protein Ki67 in mice tissues with NAT10 knockout (Figure 2G and Figure S1A).

Lentivirus‐mediated RNA interference vectors targeting NAT10 were further established in DLBCL cells, of which shNAT10#2 (NAT10 knockdown, NAT10 KD) exhibited the highest efficacy (Figure 2H,I) and was selected for subsequent functional experiments. NAT10 knockdown showed the same inhibitory effect on DLBCL cell proliferation as NAT10 knockout (Figure 2J). Moreover, overexpression efficacy of NAT10 (NAT10 OE) was verified (Figure 2K,L). Conversely, overexpression of NAT10 promoted DLBCL cell proliferation both in vitro (Figure 2M) and in vivo (Figure 2N). These evidences indicate that NAT10 is required for DLBCL proliferation.

### 2.3 Inhibition of NAT10 induced cell cycle arrest in DLBCL cells

**FIGURE 3:**
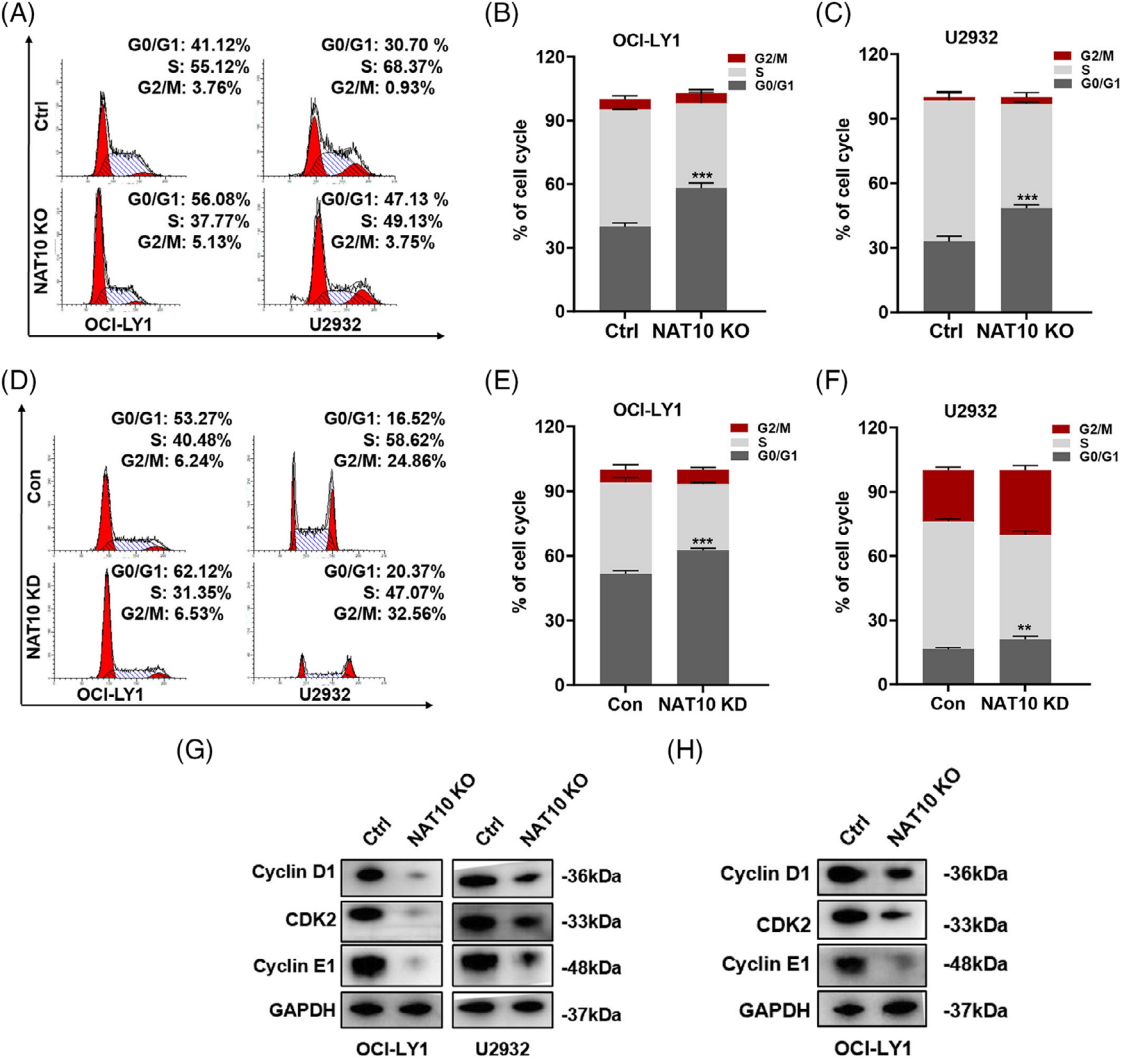
Suppression of NAT10 induced cell cycle arrest in DLBCL cells. (A–C) Knocking out NAT10 induced cell cycle arrest in OCI‐LY1 and U2932 cells, which were arrested in the G0/G1 phase. Cell cycle distribution was measured by flow cytometry. Ctrl: negative control (non‐target gRNAs), NAT10 KO: CRISPR/Cas9 gene editing system targeting deletion of NAT10 (NAT10 knockout). At least three independent experiments were conducted to obtain the data presented as mean  ±  SD. ***p < .001. *p*‐Values from unpaired two‐tailed *t*‐test. (D–F) NAT10 knockdown induced cell cycle arrest at the G0/G1 phase in OCI‐LY1 and U2932 cells. Con: control, NAT10 KD: lentivirus‐mediated NAT10 knockdown. At least three independent experiments were conducted to obtain the data presented as mean  ±  SD. **p < .01, ***p < .001. *p*‐Values from unpaired two‐tailed *t*‐test. (G) Decreased expression levels of cyclin D1, CDK2 and cyclin E1 were observed in NAT10 knockout‐treated DLBCL cells. Ctrl: negative control (non‐target gRNAs), NAT10 KO: CRISPR/Cas9 gene editing system targeting deletion of NAT10 (NAT10 knockout). (H) Decreased expression levels of cyclin D1, CDK2 and cyclin E1 were observed in tumour tissues from DLBCL xenograft model with NAT10 knockout cells. Ctrl: negative control (non‐target gRNAs), NAT10 KO: CRISPR/Cas9 gene editing system targeting deletion of NAT10 (NAT10 knockout).

Several studies have suggested that cell cycle‐associated protein inhibitors might be beneficial for DLBCL patients,^28^ suggesting cell cycle regulation plays a vital role in regulating tumour progression. The role of NAT10 in the regulation of DLBCL cell cycle was further explored. The results indicated that knockout of NAT10 led to a noticeable increase in cell counts during the G0/G1 phase, compared to the negative control group (Figure 3A–C). Consistently, knockdown of NAT10 also induced a similar cell cycle distribution (Figure 3D–F). DLBCL cells were then detected for cycle‐related proteins after NAT10 knockout. NAT10 knockout reduced cyclin D1, cyclin E1 and CDK2 protein expression (Figure 3G). Furthermore, decreased protein expression of above three proteins was also detected in the tissues isolated from DLBCL xenograft mouse model with NAT10 knockout (Figure 3H).

Due to NAT10 being enriched in apoptosis, as mentioned above, related experiments were performed. However, annexin V‐PE/7AAD apoptotic assays indicated no significant difference between NAT10 knockout and negative control in terms of cellular apoptosis in DLBCL. NAT10 was further examined for its regulatory function towards apoptosis‐related proteins, and we found that knocking out NAT10 had no significant effect on their expression (Figure S1B). Together, the above observations confirm that NAT10 promotes the proliferation of DLBCL cells and regulates their cell cycle.

### 2.4 NAT10 regulated the ac4C acetylation of SLC30A9 mRNA in DLBCL cells

**FIGURE 4:**
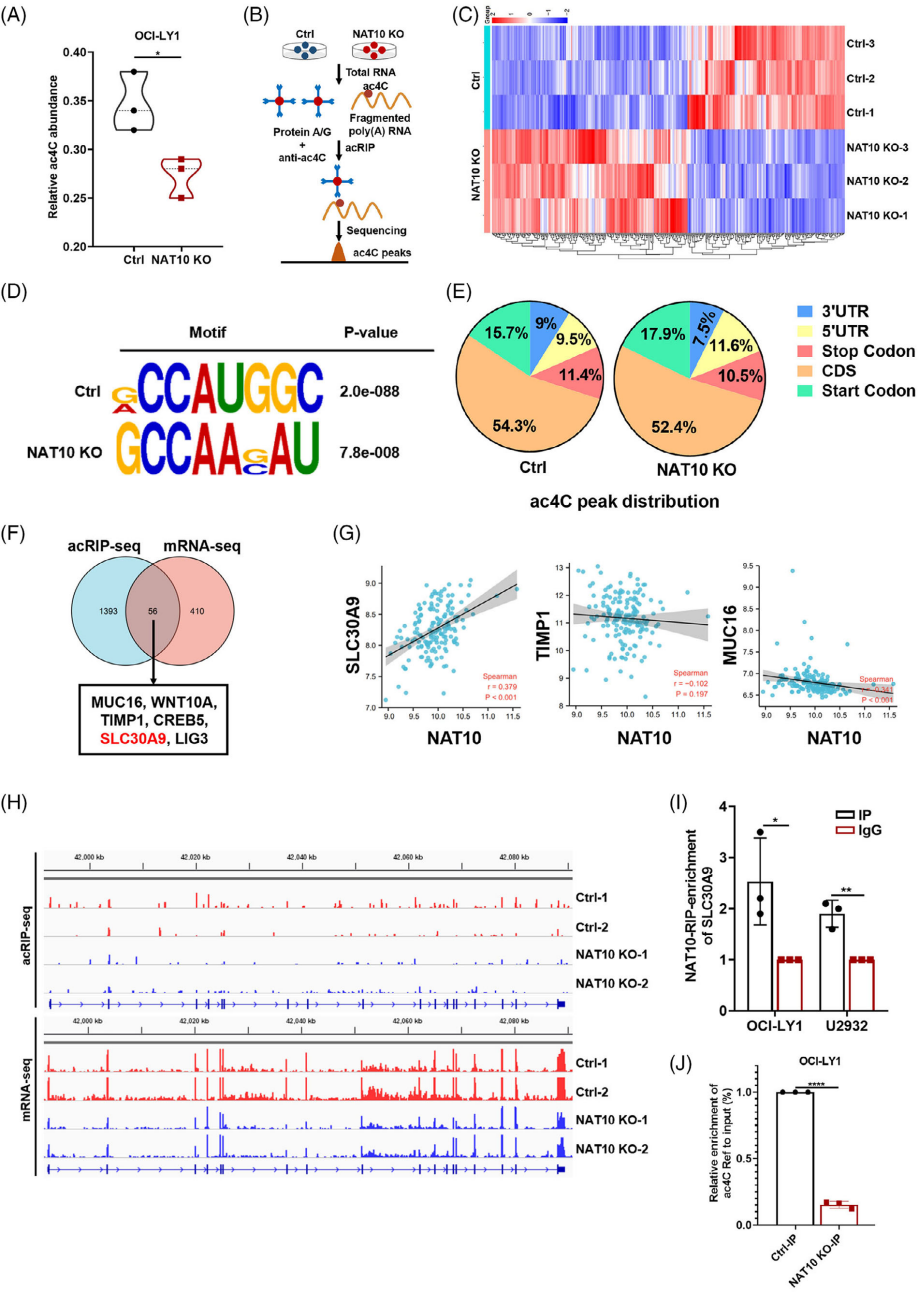
NAT10 regulated the acetylation of SLC30A9 mRNA in DLBCL cells. (A) Global ac4C abundance in OCI‐LY1 cells with negative control or NAT10 knockout, *n* = 3. Ctrl: negative control (non‐target gRNAs), NAT10 KO: CRISPR/Cas9 gene editing system targeting deletion of NAT10 (NAT10 knockout). *p < .05. *p*‐Values from unpaired two‐tailed *t*‐test. (B) The process of acRIP‐seq. (C) Differentially expressed genes between negative control and NAT10 knockout OCI‐LY1 cells from mRNA‐seq, *n* = 3. Ctrl: negative control (non‐target gRNAs), NAT10 KO: CRISPR/Cas9 gene editing system targeting deletion of NAT10 (NAT10 knockout). (D) Top consensus motif with acRIP‐seq peaks in OCI‐LY1 cells with or without NAT10 knockout. Ctrl: negative control (non‐target gRNAs), NAT10 KO: CRISPR/Cas9 gene editing system targeting deletion of NAT10 (NAT10 knockout). (E) Representative pie chart of peak distribution exhibiting the proportion of total ac4C peaks in the indicated regions. Ctrl: negative control (non‐target gRNAs), NAT10 KO: CRISPR/Cas9 gene editing system targeting deletion of NAT10 (NAT10 knockout). (F) Venn diagram showing SLC30A9 as the potential target of NAT10. (G) Scatter plots show the correlation between NAT10 and SLC30A9, CREB5, TIMP1 and MUC16. (H) Integrative Genomics Viewer (IGV) tracks displaying acRIP‐seq and mRNA‐seq reads distribution in SLC30A9 mRNA. (I) The bound and interacted relationship between NAT10 and SLC30A9 mRNA in OCI‐LY1 and U2932 cells confirmed using the RIP qRT‐PCR assay. Results are presented as mean  ±  SD from three independent experiments. *p < .05, **p  <  .01. *p*‐Values from unpaired two‐tailed *t*‐test. (J) acRIP‐qPCR displaying ac4C enrichment in SLC30A9 mRNA in negative control and NAT10 knockout cells. Ctrl: negative control (non‐target gRNAs), NAT10 KO: CRISPR/Cas9 gene editing system targeting deletion of NAT10 (NAT10 knockout). ****p < .0001. *p*‐Values from unpaired two‐tailed *t*‐test.

Given that NAT10 is characterized as acetyltransferase in ac4C modification,^29^ we further explored the role of NAT10‐mediated ac4C modification in DLBCL. LC‐MS/MS was used to quantify the overall ac4C level in NAT10 knockout DLBCL cells. The results revealed that NAT10 knockout induced a reduction in global levels of ac4C, as shown in Figure 4A. Subsequently, acetylated RNA immunoprecipitation sequencing (acRIP‐seq) (Figure 4B) and mRNA‐seq were performed in OCI‐LY1 cells with NAT10 knockout. The mRNA‐seq screened 467 differentially expressed mRNAs after NAT10 knockout, of which 209 were downregulated and 258 were upregulated (Figure 4C). Based on the transcriptome expression, acRIP‐seq was conducted to determine the distribution and transition of ac4C in the OCI‐LY1 negative control (Ctrl) and NAT10 knockout (NAT10 KO) cells. The typical consensus sequence motif for ac4C is illustrated in Figure 4D. According to our acRIP‐seq data, most of the ac4C peaks in DLBCL cells were distributed in the coding sequence (CDS) of mRNA transcripts (Figure 4E), which indicated that NAT10 might directly regulate mRNA expression in DLBCL cells.

Through the combined analysis of mRNA‐seq and acRIP‐seq, 56 genes with differentially expressed mRNA expression and differentially ac4C peak abundance were screened, with 43 upregulated and 13 downregulated. Given that the overall level of ac4C decreased after NAT10 knockout, we supposed that targets might be included in the 13 downregulated genes. According to GO enrichment gene function, a total of six potential targets of NAT10 in DLBCL were identified, including SLC30A9, CREB5, TIMP1, MUC16, LIG3 and WNT10A (Figure 4F). Among them, SLC30A9 exhibited a significant positive correlation with NAT10 expression and was selected for further study (Figure 4G). mRNA‐seq and acRIP‐seq data showed that knockout of NAT10 could significantly downregulate SLC30A9 mRNA level and reduce the enrichment of ac4C peaks on SLC30A9 mRNA CDS region (Figure 4H and Figure S1C). RIP qRT‐PCR assays demonstrated a significant binding and interaction of SLC30A9 mRNA to NAT10 (Figure 4I). Moreover, acRIP‐qPCR analysis revealed that SLC30A9 mRNA was associated with significantly decreased ac4C abundance upon NAT10 knockout in DLBCL cells (Figure 4J), which was consistent with the sequencing analysis. The results presented above suggest that NAT10 appears to be important in regulating SLC30A9 mRNA acetylation in DLBCL.

### 2.5 NAT10 acetylated SLC30A9 mRNA at K290A and G641E sites in ac4C‐dependent manner

**FIGURE 5:**
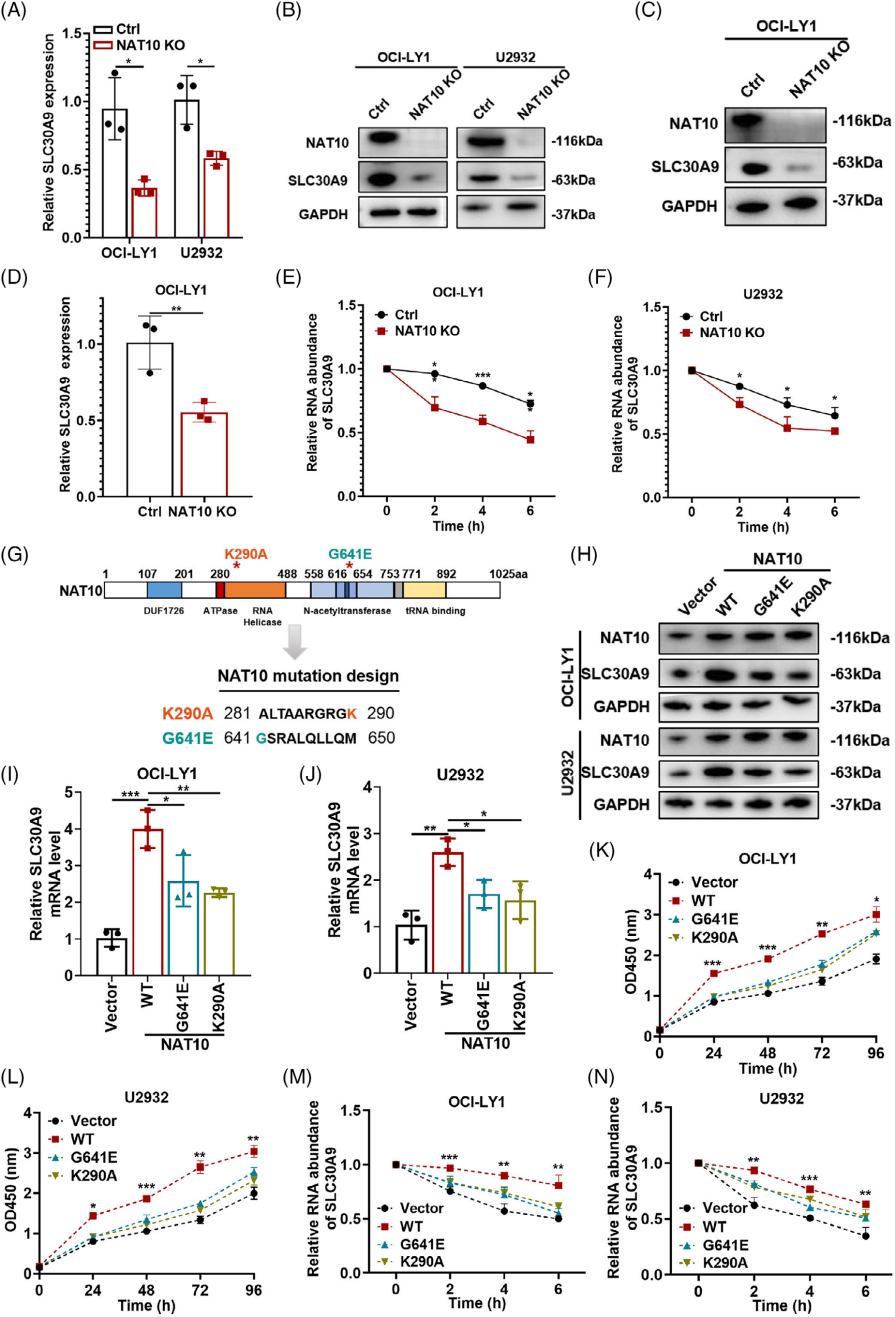
NAT10 acetylated SLC30A9 at K290A and G641E sites in ac4C‐dependent manner. (A) qRT‐PCR of SLC30A9 expression in negative control or NAT10 knockout DLBCL cells. Ctrl: negative control (non‐target gRNAs), NAT10 KO: CRISPR/Cas9 gene editing system targeting deletion of NAT10 (NAT10 knockout). Results are presented as mean  ±  SD from three independent experiments. *p  <  .05. *p*‐Values from unpaired two‐tailed *t*‐test. (B) Western blot of SLC30A9 expression in negative control or NAT10 knockout DLBCL cells. Ctrl: negative control (non‐target gRNAs), NAT10 KO: CRISPR/Cas9 gene editing system targeting deletion of NAT10 (NAT10 knockout). (C) Western blot analyses of SLC30A9 expression in tumour tissues obtained from the NAT10 knockout subcutaneous tumour. Ctrl: negative control (non‐target gRNAs), NAT10 KO: CRISPR/Cas9 gene editing system targeting deletion of NAT10 (NAT10 knockout). (D) qRT‐PCR analyses of SLC30A9 expression in tumour tissues obtained from the NAT10 knockout subcutaneous tumour. Ctrl: negative control (non‐target gRNAs), NAT10 KO: CRISPR/Cas9 gene editing system targeting deletion of NAT10 (NAT10 knockout). Results are presented as mean  ±  SD from three independent experiments. **p  <  .01. *p*‐Values from unpaired two‐tailed *t*‐test. (E and F) The impression of NAT10 on SLC30A9 mRNA stability confirmed by the RNA stability assay. Data are shown as the mean ± SD of at least three independent experiments. *p < .05, ***p < .001. *p*‐Values from unpaired two‐tailed *t*‐test. (G) Representation of NAT10 with its known domains and schematic diagram of the design of NAT10 mutant. The G641E mutation and K290A mutation are asterisked and respectively indicated in orange and green. (H) The expression of SLC30A9 is examined after transfection of plasmids containing NAT10 sequence by western blot. (I and J) The expression of SLC30A9 is examined after transfection of plasmids containing NAT10 sequence by qRT‐PCR. Data are shown as the mean ± SD of at least three independent experiments. *p < .05, **p  <  .01, ***p < .001. *p*‐Values from unpaired two‐tailed *t*‐test. (K and L) CCK‐8 kit is utilized to determine the proliferation level in OCI‐LY1 and U2932 cells transfected by empty vector, NAT10 wild‐type plasmid (WT) and NAT10 mutant plasmid (G641E and K290A), respectively. Data are shown as the mean ± SD of at least three independent experiments. *p < .05, **p  <  .01, ***p < .001. *p*‐Values from two‐way ANOVA with Sidak correction. (M and N) The mRNA half‐life of SLC30A9 in OCI‐LY1 and U2932 cells transfected with empty vector or wild‐type NAT10 (WT) or NAT10 mutant (G641E and K290A). Data are shown as the mean ± SD of at least three independent experiments. **p < .01, ***p < .001. *p*‐Values from unpaired two‐tailed *t*‐test.

As NAT10 could regulate SLC30A9 mRNA ac4C level, the regulatory mechanism underlying mRNA acetylation of SLC30A9 was explored further. Based on western blotting and qRT‐PCR, it was shown that the expression of SLC30A9 was decreased after knockout of NAT10 both in vitro (Figure 5A,B) and in vivo (Figure 5C,D and Figure S1D), and increased when NAT10 was overexpressed (Figure S2A,B). Given that RNA ac4C modification has been reported to increase mRNA stability and translation,^15^, ^16^, ^30^ RNA stability assay was applied. The results revealed that SLC30A9 mRNA half‐life was markedly shorter in NAT10 knockout DLBCL cells, compared to negative control (Figure 5E,F).

To investigate whether NAT10 acetylated SLC30A9 in an ac4C‐dependent manner, we overexpressed NAT10 wild‐type (WT) and mutant plasmids lacking functional RNA helicase (K290A) and acetyltransferase (G641E) sites in OCI‐LY1 and U2932 cells (Figure 5G), which were previously reported to destroy its RNA acetyltransferase function.^19^, ^31^, ^32^, ^33^ SLC30A9 protein and mRNA levels are elevated by NAT10 WT compared to NAT10 mutants (G641E and K290A) (Figure 5H–J). As previously mentioned, overexpression of NAT10 promoted DLBCL cell proliferation. RNA‐seq upon overexpression of NAT10 WT and NAT10 mutants was performed to further prove whether the enzymatic function is important in the regulation of proliferation. GO enrichment analysis showed that NAT10 participated in cell cycle and cell death (Figure S2C). NAT10 WT also stimulated the proliferative capacity of DLBCL cells (Figure 5K,L). Compared to two mutants, re‐expression of NAT10 WT in NAT10 knockout cells effectively restored proliferation level, suggesting that the RNA ac4C activity of NAT10 is critical for its role in DLBCL (Figure S2D). In addition, SLC30A9 mRNA stability was increased in both OCI‐LY1 and U2932 cells with NAT10 WT, but not with NAT10 mutants (Figure 5M,N). Consequently, the inhibition of the acetylation activity of NAT10 significantly impaired the expression and stability of SLC30A9. It indicates that NAT10 acetylates SLC30A9 at K290A and G641E in an ac4C‐dependent manner.

### 2.6 NAT10 promoted DLBCL progression by regulating SLC30A9‐mediated activation of AMPK/mTOR signalling

SLC30A9 is a highly conserved zinc (Zn^2+^) transporter in the solute carrier family 30 (SLC30).^34^ Several further studies were conducted to assess the function of SLC30A9 in DLBCL. The mRNA expression of SLC30A9 was further verified using the GEO database (GSE83632) (Figure S2E). Based on the GEO database (GSE32918), the higher expression of SLC30A9 in DLBCL was associated with poor outcomes (Figure S2F). We further investigated the SLC30A9 protein expression level by IHC in primary DLBCL samples from SPH. Compared with reactive hyperplasia lymphoid, DLBCL tissues displayed increased SLC30A9 expression (Figure S2G, *p* = .0264). Moreover, we explored the correlation between NAT10 and SLC30A9 in DLBCL tissue samples from the same patients. Consistent with the previous results, SLC30A9 was positively correlated with NAT10 (Figure S2H).

**FIGURE 6:**
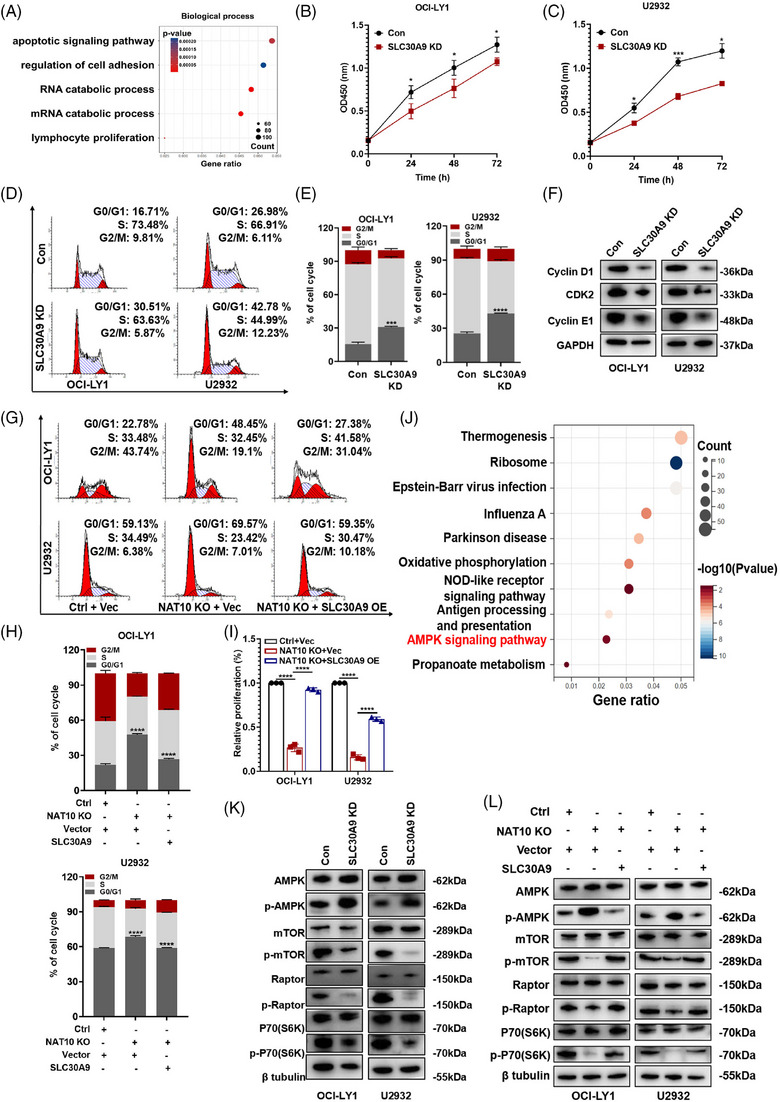
NAT10 facilitated DLBCL progression by regulating SLC30A9‐mediated activation of AMPK signalling. (A) GO analysis of the differentially expressed genes in SLC30A9‐deficient OCI‐LY1 cells from RNA‐seq. (B and C) SLC30A9 knockdown decreased cellular proliferative activity in OCI‐LY1 and U2932 cells. Con: control, SLC30A9 KD: lentivirus‐mediated SLC30A9 knockdown. At least three independent experiments were conducted to obtain the data presented as mean  ±  SD. *p < .05, ***p < .001. *p*‐Values from two‐way ANOVA with Sidak correction. (D and E) SLC30A9 depletion induced cell cycle arrest at the G0/G1 phase in OCI‐LY1 and U2932 cells. Cell cycle distribution was detected using flow cytometry. Con: control, SLC30A9 KD: lentivirus‐mediated SLC30A9 knockdown. At least three independent experiments were conducted to obtain the data presented as mean  ±  SD. ***p < .001, ****p < .0001. *p*‐Values from unpaired two‐tailed *t*‐test. (F) Decreased expression level of cyclin D1, CDK2 and cyclin E1 was observed in SLC30A9 knockdown‐treated DLBCL cells. Con: control, SLC30A9 KD: lentivirus‐mediated SLC30A9 knockdown. (G and H) OCI‐LY1 and U2932 cells were transfected with negative control or NAT10 knockout sgRNA, together with an empty vector or SLC30A9‐encoding lentivirus as indicated. After drug selection, those co‐transfected cells were seeded into six‐well plates for cell cycle assays. Ctrl: negative control (non‐target gRNAs), NAT10 KO: CRISPR/Cas9 gene editing system targeting deletion of NAT10 (NAT10 knockout), Vec: empty vector, SLC30A9 OE: SLC30A9 overexpression. Data are shown as the mean ± SD of at least three independent experiments. ****p < .0001. *p*‐Values from unpaired two‐tailed *t*‐test. (I) OCI‐LY1 and U2932 cells were transfected with negative control or NAT10 knockout sgRNA, together with an empty vector or SLC30A9‐encoding lentivirus as indicated. After drug selection, those co‐transfected cells were seeded into 96‐well plates for cell proliferation assays. Ctrl: negative control (non‐target gRNAs), NAT10 KO: CRISPR/Cas9 gene editing system targeting deletion of NAT10 (NAT10 knockout), Vec: empty vector, SLC30A9 OE: SLC30A9 overexpression. Data are shown as the mean ± SD of at least three independent experiments. ****p < .0001. *p*‐Values from unpaired two‐tailed *t*‐test. (J) KEGG analysis of SLC30A9 associated signalling pathway based on the RNA‐seq data. (K) The levels of AMPK, p‐AMPK, mTOR, p‐mTOR, Raptor, p‐Raptor, p70(S6K) and p‐p70(S6K) were detected by western blot after SLC30A9 knockdown. Con: control, SLC30A9 KD: lentivirus‐mediated SLC30A9 knockdown. (L) The inhibition of the AMPK‐mTOR‐p70(S6K) signalling pathway by NAT10 was reversed with SLC30A9 overexpression. Ctrl: negative control (non‐target gRNAs), NAT10 KO: CRISPR/Cas9 gene editing system targeting deletion of NAT10 (NAT10 knockout), Vec: empty vector, SLC30A9 OE: SLC30A9 overexpression.

Functional experiments were conducted to investigate the role of SLC30A9 in DLBCL. The effective regulation of SLC30A9 expression was demonstrated (Figure S2I,J), with shSLC30A9#1 (SLC30A9 knockdown, SLC30A9 KD) exhibiting the highest efficacy. The function of SLC30A9 in DLBCL cells was evaluated by enrichment analysis of DEGs (Figure S2K) following RNA‐seq. GO enrichment analysis indicated that SLC30A9 was related to apoptotic, mRNA catabolic, as well as proliferation processes (Figure 6A). Consistent with the effects of NAT10 knockout, SLC30A9 knockdown (SLC30A9 KD) also markedly reduced cell proliferation (Figure 6B,C) and arrested cell cycle process (Figure 6D,E) in DLBCL cells. Knockdown of SLC30A9 resulted in a reduction in cyclin D1, CDK2 and cyclin E1 expression, inducing G0/G1 phase cycle arrest (Figure 6F). Conversely, overexpression of SLC30A9 also facilitated DLBCL cell proliferation (Figure S2L). Moreover, it is noteworthy that the inhibitory effects of NAT10 knockout on cell cycle arrest (Figure 6G,H) and cell proliferation (Figure 6I) can be primarily rescued by forced expression of SLC30A9.

KEGG enrichment analysis indicated that SLC30A9 was functionally enriched in the AMP‐activated protein kinase (AMPK) signalling pathway (Figure 6J). It has been demonstrated that AMPK signalling pathways are essential for cell growth, autophagy and metabolism.^35^ As shown in Figure 6K, SLC30A9 knockdown inhibited the phosphorylation of Raptor, p70(S6K) and mTOR by positively regulating AMPK and its phosphorylated form. Further studies were carried out to determine how NAT10 modulates AMPK signalling pathway. NAT10 knockout increased AMPK phosphorylation and decreased Raptor, mTOR and p70(S6K) phosphorylation (Figure S2M). Additionally, enforced SLC30A9 expression partially reversed inhibition by NAT10 knockout (Figure 6L). According to these findings, NAT10 appears to regulate AMPK‐mTOR‐p70(S6K) signalling pathway via SLC30A9 to promote the growth of DLBCL.

### 2.7 NAT10 inhibitor remodelin exerted anti‐tumour effects and enhanced the efficacy of ibrutinib in DLBCL

**FIGURE 7:**
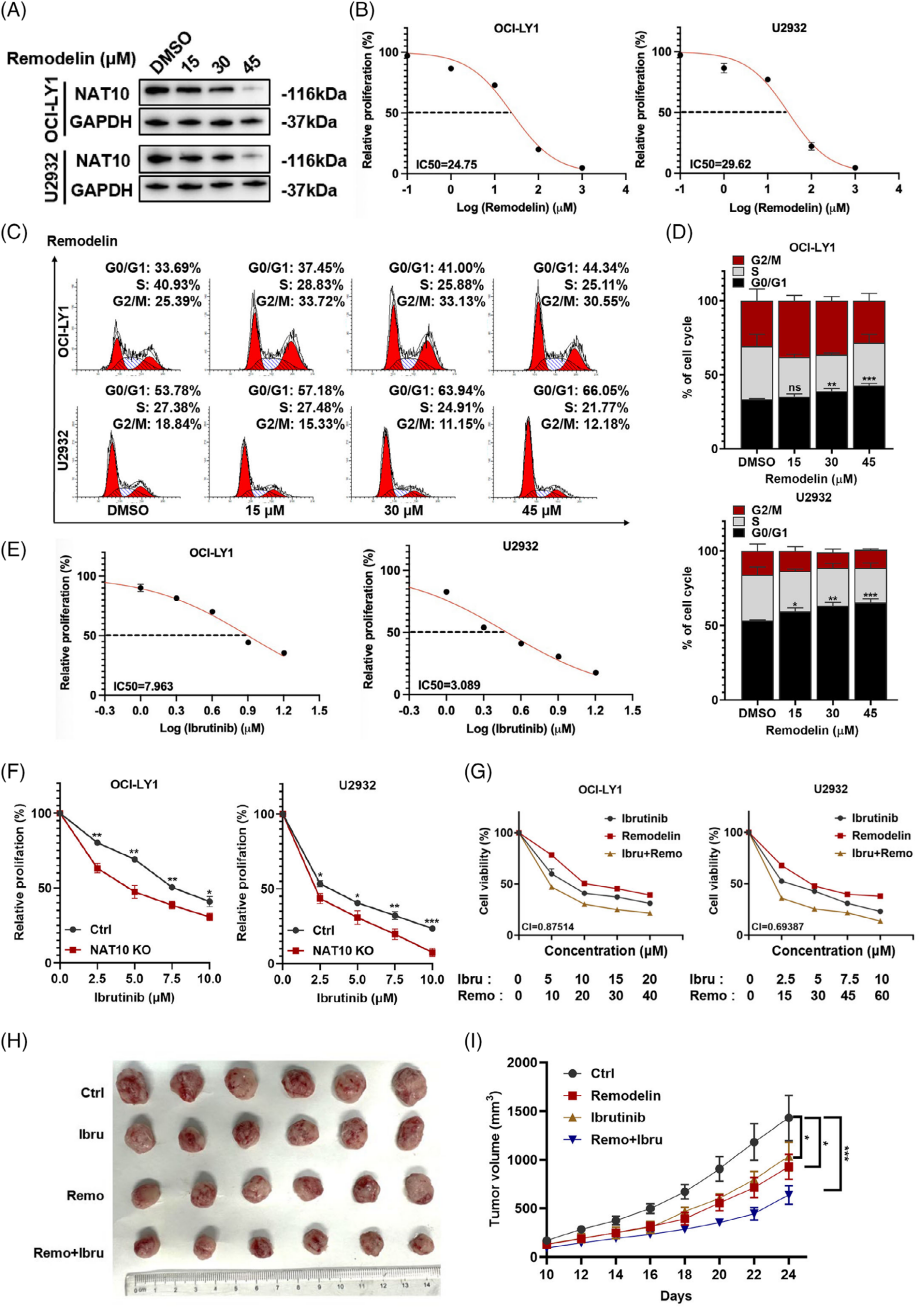
NAT10 inhibitor remodelin exerted anti‐tumour effects and enhanced the efficacy of ibrutinib in DLBCL. (A) The effect of remodelininhibited NAT10 was evaluated by western blot analysis. (B) The IC50 of remodelin in DLBCL cells. (C and D) Treatment with different concentrations of remodelin for 48 h induced G0/G1 phase arrest in DLBCL cells. Data are shown as the mean ± SD of at least three independent experiments. *p < .05, **p < .01, ***p < .001, ns: no significance. *p*‐Values from unpaired two‐tailed *t*‐test. (E) The IC50 of ibrutinib in DLBCL cells. (F) OCI‐LY1 and U2932 cells transfected with a negative control or NAT10 knockout were treated with ibrutinib at the indicated concentrations for 48 h before being subjected to CCK‐8 assay. Ctrl: negative control (non‐target gRNAs), NAT10 KO: CRISPR/Cas9 gene editing system targeting deletion of NAT10 (NAT10 knockout). Data are shown as the mean ± SD of at least three independent experiments. *p < .05, **p < .01, ****p  <  .0001. *p*‐Values from unpaired two‐tailed *t*‐test. (G) The use of remodelin increases the chemosensitivity of DLBCL cell lines to ibrutinib. CI: combination index. (H and I) A mice xenograft model was constructed to investigate the efficacy of the drug combination in vivo (*n* = 6). *p < .05, ***p < .001. *p*‐Values from two‐way ANOVA with Sidak correction.

Remodelin, an inhibitor of NAT10, was used to evaluate the effect of NAT10 in DLBCL. Recent studies revealed that remodelin inhibited tumour cell proliferation and enhanced cell cycle arrest.^36^ We verified the inhibitory effect of remodelin on NAT10 in DLBCL cells (Figure 7A,B). Remodelin inhibited G0/G1 phase progression according to flow cytometry analysis (Figure 7C,D). This indicates that remodelin exerts its anti‐tumour effects through antiproliferation and cell cycle arrest.

Bruton's tyrosine kinase (BTK) participates in oncogenic signalling and significantly affects malignant B cells.^37^ As a BTK inhibitor, ibrutinib prevents BTK from kinase activity^38^ and has demonstrated remarkable clinical effectiveness.^39^ Despite this, ibrutinib treatment leads to insufficient responses as well as drug resistance.^40^ Consequently, we investigated the potential impact of NAT10 on DLBCL cells responding to ibrutinib via CCK‐8 assays (Figure 7E). Remarkably, the cell proliferation of NAT10 knockout DLBCL cell lines was markedly reduced (Figure 7F). By combining ibrutinib with remodelin, we evaluated the cell proliferation levels of OCI‐LY1 and U2932 to further investigate the effects of NAT10 on ibrutinib in vitro. As shown in Figure 7G and Figure S3A, the combination of ibrutinib with remodelin could reduce the cell proliferation of OCI‐LY1 and U2932. A mice xenograft model was then constructed to investigate the efficacy of remodelin and the drug combination in vivo. The result indicated that remodelin inhibited the growth of mice xenograft model. Compared with remodelin monotherapy, combination treatment significantly suppressed the growth of DLBCL cells (Figure 7H,I).

Single agent target of rapamycin (a mTOR inhibitor) treatment has shown promising efficacy in DLBCL.^41^ Besides, we also performed the CCK8 assay to explore the effect of rapamycin in combination with remodelin or ibrutinib as shown in Figure S3B, and found that combination treatment with ibrutinib or remodelin could suppress proliferation of DLBCL cells compared to rapamycin monotherapy.

## 3 DISCUSSION

Epigenome dysregulation leads to aberrant transcriptional programs that promote cancer development and progression, and has now become a vital area for early diagnosis and treatment of cancer.^30^, ^42^ After its transcription, RNA can be modified by more than 170 chemically distinct modifications (epitranscriptome). Deposition of RNA modifications in the tumour environment is dynamic, which allows tumour cells to respond rapidly to environmental changes and adapt to their microenvironment to ensure their proliferation. This suggests that RNA modifications might have significant impact on tumours.^43^, ^44^ Recent studies have revealed that aberrant ac4C modifications could also be detected in various tumour types.^16^, ^30^ The presence of ac4C is essential to enhance the stability of RNA and maintain the fidelity of protein translation.^45^, ^46^

Previous studies showed that ac4C modification is mainly located in non‐coding RNAs.^33^, ^47^, ^48^ Arango et al. initially reported the existence of ac4C modification, which could promote RNA translation and increase RNA stability.^15^ Abundant evidence indicates that ac4C modification plays a prominent role in human diseases, especially in tumorigenesis. As the only known “writer” protein for ac4C synthesis, NAT10 is upregulated in a variety of malignant tumours such as gastric cancer, bladder cancer, colorectal cancer, bladder cancer and involved in poor prognoses.^17^, ^19^, ^30^, ^49^ Our results demonstrated that elevated expression of NAT10 in DLBCL, and high levels of NAT10 expression contributed to poor outcomes in DLBCL patients. Functionally, the tumorigenic role of NAT10 was revealed both in vitro and in vivo that investigated the cell proliferation and cell cycle regulation of DLBCL cells. Collectively, these findings suggest an oncogenic role of NAT10 in DLBCL.

To understand the underlying mechanism of NAT10 on regulating ac4C modification in DLBCL, mRNA‐seq and acRIP‐seq were conducted, and SLC30A9 was found as an intermediate target. SLC39A and SLC30A are two families of zinc transporters.^50^ Zinc also plays a prominent role in cellular signalling, protein synthesis and regulation of gene expression. As a result of our study, KEGG enrichment analysis revealed that SLC30A9 might play a role in regulating AMPK signalling pathway in DLBCL. Further investigation also revealed that NAT10 could enhance SLC30A9 expression by ac4C acetylation on SLC30A9 mRNA and regulating AMPK‐mTOR‐p70(S6K) signalling pathway. Recently, increasing evidence has suggested that NAT10 inhibitor remodelin can regulate cell proliferation,^19^ cell apoptosis and cell cycle.^18^ More importantly, we observed that remodelin inhibited cell growth, which revealed the antiproliferative effect of remodelin in DLBCL. BTK inhibitor ibrutinib has shown comparable efficacy in the treatment of haematological malignancies in recent years.^51^, ^52^, ^53^ In addition, we observed that the combination of ibrutinib and remodelin was more effective in inhibiting tumour growth than either ibrutinib or remodelin alone in vivo xenograft model.

Meanwhile, there are still some limitations in our study. Firstly, our xenograft models were used with cell line‐derived xenograft, patient‐derived xenograft (PDX) models of DLBCL would be more helpful in comprehending the role of NAT10 in DLBCL progression. Secondly, according to the current research, transcription factor (TF) could activate the transcription of gene and thus promote the expression level of gene in tumours,^54^, ^55^ which provides some insights into the upregulation of NAT10 in DLBCL. Future exploration of the underlying mechanism is required.

**FIGURE 8:**
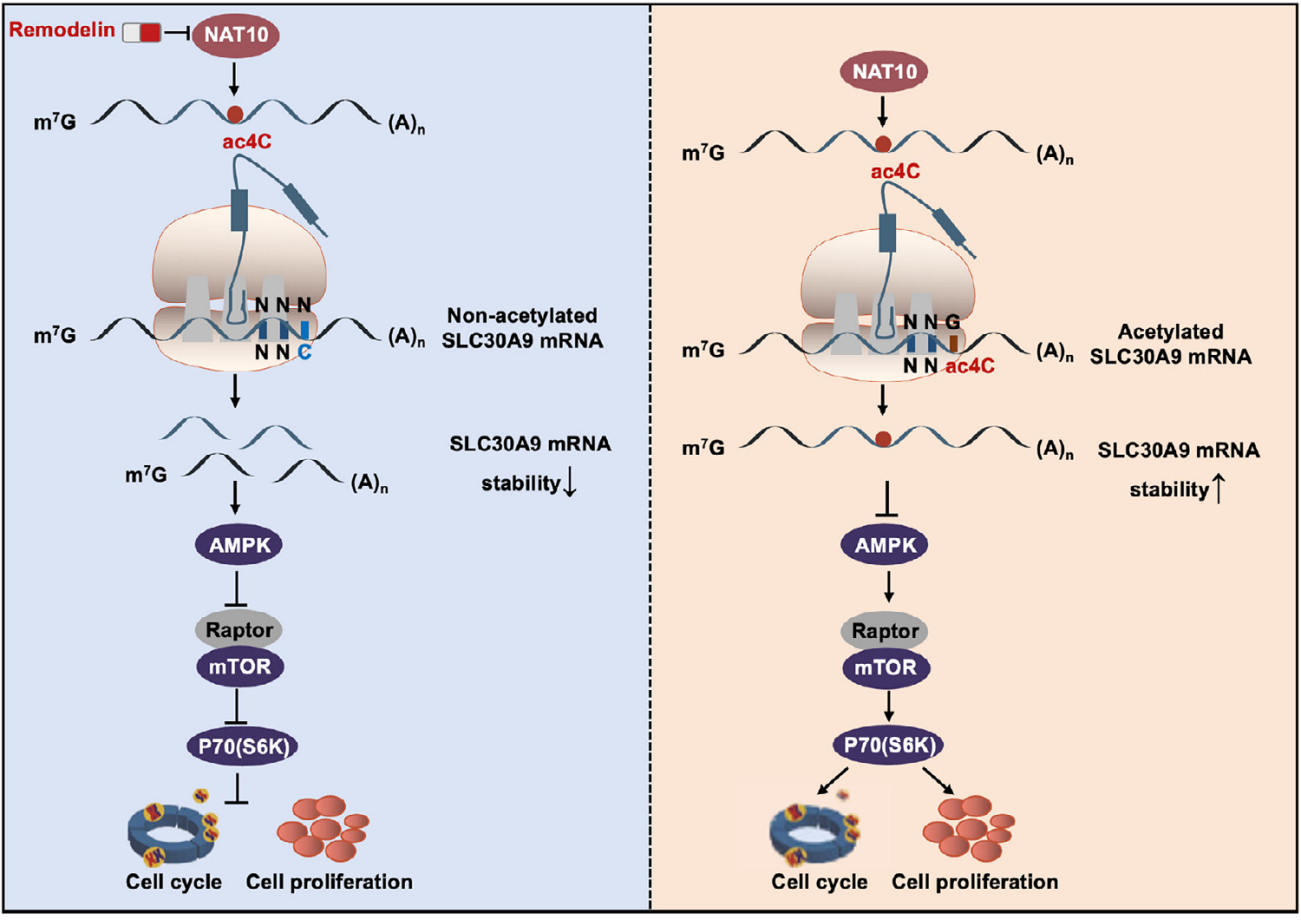
Mechanism diagram summarizing that NAT10 facilitates progression of DLBCL by regulating ac4C modification of SLC30A9.

In summary, we revealed that NAT10 promoted progression of DLBCL via mediating ac4C acetylation of SLC30A9 mRNA and regulating AMPK‐mTOR‐p70(S6K) signalling pathway (Figure 8). Furthermore, NAT10 inhibitor remodelin could enhance the response sensibility of DLBCL cells to BTK inhibitor ibrutinib. By the application of remodelin, NAT10 might provide a promising treatment strategy for DLBCL.

## 4 MATERIALS AND METHODS

### 4.1 CRISPR/Cas9‐mediated knockout of NAT10

CRISPR/Cas9 genomic editing system was used to create NAT10 knockout (NAT10 KO) cells. The stable lentivirus vectors were produced and packaged by OBiO Technology (Shanghai, China). DLBCL cells transfected with lentivirus and puromycin selection (5 µg/mL) were applied after 3 days, then the knockout cells were further isolated as described previously.^56^ The gRNAs target sites for NAT10 deletion were followed: pLenti‐U6‐spgRNA (NAT10)‐CMV‐Puro‐P2A‐3Flag‐spCas9.

### 4.2 Acetylated RNA immunoprecipitation sequencing (acRIP‐seq) and data analysis

CloudSeq Inc. (Shanghai, China) provided the acRIP‐seq service. In OCI‐LY1 cells with NAT10 knockout, total RNA was extracted, rRNA was depleted with Ribo‐zero (Illumina), and immunoprecipitation was performed using the GenSeqTM ac4C‐IP Kit (GenSeq Inc., China). In brief, RNA was randomly sliced to approximately ~200 nt and co‐incubated with protein A/G beads that had been mixed with ac4C antibody and rotated at 4°C for 4 h. Subsequently, the RNA was eluted and purified. RNA librarie were prepared using the NEBNext Ultra II Directional RNA Library Prep Kit from IP and input samples. Libraries were characterized by Agilent 2100 Bioanalyzer and sequenced on the Novaseq 6000 platform. MACS software was used to identify the acetylated sites on RNA (peaks). DiffReps was used to determine differentially acetylated sites. Homemade scripts were used to identify and select the peaks overlapping with exons of mRNA.

### 4.3 Detection of ac4C by LC‐MS/MS

In order to determine the ac4C/C ratio in total RNA and mRNA, Cloud‐Seq Biotech Ltd. performed LC‐MS/MS analysis. Briefly, the RNA was digested into nucleosides under 37°C conditions using buffer, S1 nuclease, alkaline phosphatase and phosphodiesterase. Following digestion, the mixture was extracted with chloroform and analyzed by UPLC‐ESI‐MS/MS system. Nucleosides were detected via a QTRAP 6500 LC‐MS/MS platform from AB Sciex. The chromatographic conditions were as follows: ACQUITY HSS T3 column was used, i.d. 2.1 × 100 mm, 1.8 µm; mobile phase A and B was 2 mM ammonium bicarbonate/ultra‐pure water and 2 mM ammonium bicarbonate/methanol, respectively; sample size was 10 µL and column temperature was 40°C. Overall ac4C levels are expressed as a percentage of ac4C/rC.

### 4.4 RNA stability assay

Six‐well plates were used to culture the cells for the RNA stability assay. Following this, 5 µg/mL actinomycin D (#A9415, Sigma) was applied. A total RNA sample was isolated from the cells after 0, 2, 4 and 6 h. To quantify SLC30A9 mRNA abundance, qRT‐PCR was performed.

### 4.5 Statistical analysis

Statistical analysis was performed using SPSS 23.0 and Graphpad Prism 8.0. The results were expressed as the mean ± SD. All results were obtained by three independent experiments and expressed as mean ± SD. *χ* ^2^ tests was used for contingency tables. The statistical significance was evaluated using unpaired two‐tailed *t*‐test or two‐way ANOVA as appropriate. Survival analysis was measured by log‐rank tests. Statistical significance was denoted by *p* < .05.

## AUTHOR CONTRIBUTIONS

**Xin Wang** and **Xiangxiang Zhou** designed and conducted experiments. **Mengfei Ding** and **Zhuoya Yu** performed the experiments and analyzed the data. **Mengfei Ding** and **Zhuoya Yu** conducted the animal experiments and collected clinical samples. **Mengfei Ding; Zhuoya Yu; Xiangxiang Zhou** and **Xin Wang** edited the paper. All authors reviewed and approved the final manuscript.

## CONFLICT OF INTEREST STATEMENT

The authors declare that they have no conflicts of interest.

## FUNDING INFORMATION

National Natural Science Foundation, Grant Numbers: 82270200, 82170189, 82070203, 81800194, 81770210; Key Research and Development Program of Shandong Province, Grant Number: 2018CXGC1213; China Postdoctoral Science Foundation, Grant Number: 2021T1404223; Translational Research Grant of NCRCH, Grant Numbers: 2021WWB02, 2020ZKMB01; Shandong Provincial Natural Science Foundation, Grant Number: ZR2021YQ51; Taishan Scholars Program of Shandong Province, Grant Number: tspd20230610; Shandong Provincial Engineering Research Center of Lymphoma; Academic Promotion Programme of Shandong First Medical University, Grant Number: 2019QL018

## ETHICS STATEMENT

This study was approved by the Medical Ethical Committee of Shandong Provincial Hospital. All samples were obtained with informed consent in accordance with the Declaration of Helsinki. All animal experimental procedures were performed in accordance with the protocols approved by the Institutional Animal Care and Research Advisory Committee of Shandong Provincial Hospital. Ethics approval and consent to participate number: 2021‐091.

## Supporting information

Supporting Information

Supporting Information

## Data Availability

The data generated in this study are available within the article and its Supporting Information files.
