# Supplementary material for: N‐acetyltransferase 10 facilitates tumorigenesis of diffuse large B‐cell lymphoma by regulating AMPK/mTOR signalling through N4‐acetylcytidine modification of SLC30A9
Source: Clin Transl Med. 2024 Jul 3;14(7):e1747. doi: 10.1002/ctm2.1747 (PMC11222071; doi:10.1002/ctm2.1747)
Supplement: Supplementary file 1 — Supporting Information [file CTM2-14-e1747-s002.docx]

**Supplementary Materials and Methods**

**Patient and healthy donor samples**

On the basis of the Declaration of Helsinki, the Medical Ethics Committee of Shandong Provincial Hospital (SPH) approval was obtained for this study. We enrolled 104 patients with newly identified DLBCL and 20 patients with reactive hyperplasia lymphoid (RHL) from SPH. Based on the classification of WHO ^1^, the histological diagnosis was determined. Briefly, peripheral venous blood of healthy donors was extracted with anticoagulation tube, each tube was about 10ml. Then peripheral blood mononuclear cells (PBMCs) were extracted using the Ficoll-Hypaque density gradient centrifugation method (TBD Science, Tianjin, China) for further treatment, or stored in the refrigerator at -80℃ for later use. A CD19^+^ magnetic microbeads kit (Miltenyi Biotec, Bergisch Gladbach, Germany) was used to purify normal B cells from isolated PBMCs ^2^.

**Cell culture and reagents**

Human DLBCL cell lines OCI-LY1, OCI-LY3, OCI-LY8, OCI-LY10 and U2932 were used in this study. OCI-LY1, OCI-LY3 and OCI-LY8 were cultured in IMDM medium (Gibco, MD, USA). OCI-LY10 and U2932 were cultured in 1640 medium (Gibco). All cells were cultivated at 37 °C with 5% CO_2_ for the entire incubation period. Cell fluid was changed every other day, cell passage was carried out in time according to cell growth status and cell density (about 2-3 days), and cells with better growth status were selected for follow-up experiments. Rapamycin (S1842) was purchased from the Beyotime (Shanghai, China). Ibrutinib (S2680) and remodelin (S7641) were purchased from the Selleck.

**Immunohistochemistry (IHC)**

The number of 104 new DLBCL specimens was evaluated. The IHC staining was performed as formerly described ^6^. The results of IHC were assessed and scored by two independent observers with unclear information about the patients. IHC score for the protein was calculated based on the followings: IHC score = protein staining intensity (0: negative staining; 1: weak staining; 2: moderate staining; 3: strong staining) × percentage of positive cells (0: 0%; 1: 1%‐25%; 2: 26%‐50%; 3: 51%‐75%; 4: 76%‐100%). And the IHC score of 0-6 were defined as low expression, 7-12 were defined as high expression. In this assay, the following antibodies were used: NAT10 antibody (13365-1-AP, Proteintech), SLC30A9 antibody (PA5-52043, Invitrogen), Ki67 antibody (27309-1-AP, Proteintech).

**Lentivirus-mediated knockdown or overexpression of genes**

The stable knockdown of NAT10 (NAT10 KD), as well as SLC30A9 (SLC30A9 KD) and control (Con) were created by Genechem (Shanghai, China). In addition, in collaboration with Genechem, lentivirus vectors overexpressing NAT10 and SLC30A9 (NAT10 OE and SLC30A9 OE), as well as empty vector (Vec) were constructed. The company's process for designing lentivirus is as follows: for the construction of NAT10 and SLC30A9 knockdown, short hairpin RNA (shRNA) specifically targeting NAT10 and SLC30A9 was designed by the Genechem and cloned into the GV493 vector. Viral titration was as follows: shNAT10#1: 6E+8 TU/mL, shNAT10#2: 1E+9 TU/mL , shNAT10#3: 5E+8 TU/mL; shSLC30A9#1: 2E+9 TU/mL, shSLC30A9#2: 1E+9 TU/mL. shSLC30A9#3: 1E+9 TU/mL. For the construction of NAT10 or SLC30A9 overexpression, the human NAT10‐coding sequence (NM_024662.3) or SLC30A9 (NM_006345) was designed by the Genechem and cloned into the lentiviral GV492 vector. Viral titration was as follows: Lv-NAT10: 3.5E+08 TU/mL, Lv-SLC30A9: 1E+9 TU/mL. To construct stable NAT10 knockdown, SLC30A9 knockdown, NAT10 overexpression and SLC30A9 overexpression DLBCL cell lines, the manufacturer's instructions were followed for lentivirus transfected at a multiplicity of infection (MOI) =100. In order to select the stably transfected cells, puromycin was added to the culture medium (5 μg/ml, Sigma-Aldrich, USA). A 72h timeframe was used for the collection and analysis of cells. The shRNA sequences for shNAT10 and shSLC30A9 were listed in **Supplementary Table S1**.

**RNA sequencing (RNA‑seq)**

Extraction of total RNA from three stable NAT10 knockout OCI-LY1 cells and their relevant controls was performed using trizol reagent (TaKaRa, Dalian, China). mRNA-seq study was performed by CloudSeq Biotech (Shanghai, China). The rRNAs were removed from total RNA using Ribo-Zero rRNA Removal Kits based on the manufacturer's protocol. RNA libraries were constructed with the TruSequ Stranded Total RNA Library Prep Kit. Using a BioAnalyzer 2100 system, libraries were quality controlled. On an Illumina NovaSeq 6000 sequencer, sequencing was carried out. The quality control was performed by Q30 after the read pairs had been harvested. Cuffdiff software was used to get the gene level FPKM as the expression profiles of mRNA, and fold change and p-value were calculated based on FPKM, differentially expressed mRNA were identified. RNA-seq study of SLC30A9 was performed by Novogene (Beijing, China). Briefly, the sequencing libraries were generated from purified mRNA, after which were sequenced on an Illumina HiSeq platform to generate 150 bp paired-end reads. HTSeq v0.6.0 was then applied to calculate the numbers of reads and the fragments per kilobase million (FPKM). GO and KEGG analyses were performed by R language.

**Flow cytometry analysis**

A total of 1×10^6^ cells were collected from 6-well plates for relevant experiments. 70% ethanol fixation at -20 °C overnight was followed by washing the DLBCL cells with PBS. The cell cycle was measured by PI/RNase Staining Buffer (BD Biosciences, CA, USA). Cells were stained with the corresponding reagent for 15 minutes and monitored by Navios flow cytometer. The data was analyzed using Modfit software, then the proportions of each cell cycle were calculated and plotted. Based on manufacturer's protocol, Annexin V-PE/7-aminoactinomycin (7AAD) apoptosis detection assays (BD Biosciences) were used to detect apoptosis in response to the designed treatments. Using Annexin V-PE and 7AAD, cells were stained for 15 minutes in the dark and he the percentage of apoptotic cells subsequently determined by Navios flow cytometer.

**Western blot analyses**

Western blot analyses were accomplished as mentioned previously ^3^. After the treatment with chemiluminescence detection reagent (Merck Millipore, MA, USA), the chemiluminescence signal was detected by Amersham Imager 600 imaging system (General Electric, USA). ImageJ software was used for band quantification. In this assay, the following antibodies were used: NAT10 antibody (13365-1-AP, Proteintech, USA), SLC30A9 antibody (PA5-101969, Invitrogen), Caspase-9 (9508, Cell Signaling Technology), cleaved Caspase-9 (52873, Cell Signaling Technology), PARP (#9542, Cell Signaling Technology), cleaved PARP (5625, Cell Signaling Technology), CDK2 (#2546, Cell Signaling Technology), Cyclin D1 (#2922, Cell Signaling Technology), Cyclin E1 (4129T, Cell Signaling Technology), AMPK (5832T, Cell Signaling Technology), mTOR (2983T, Cell Signaling Technology), Phospho-mTOR (2974T, Cell Signaling Technology), Raptor (2280T, Cell Signaling Technology), p-Raptor(Ser792) (2083T, Cell Signaling Technology), P70(S6K) (14485–1-AP, Proteintech), Phospho-p70(S6K) (9234T, Cell Signaling Technology).

**RNA extraction and qRT-PCR**

For probing the function and RNA expression level of NAT10, total RNA was derived using the Trizol reagent (TaKaRa, Dalian, China). As per the protocol, a reverse transcription reagent (R323-01, Vazyme) was used to process the reverse-transcribed reaction. Assays were performed using LightCycler480 II with SYBR Green Master Mix (Q712, Vazyme). Relative gene expression was normalized to GAPDH mRNA expression and relative quantification was analyzed using the 2^−ΔΔCT^ method. The primer sequences are presented in **Supplementary Table S2**. The specificity of the primers used in qRT-PCR assays was confirmed by Primer-BLAST tool.

**RNA Immunoprecipitation (RIP)-qPCR**

The RIP-qPCR assay was conducted with the RIP-kit (Bes5101, BersinBio, China) as directed by the manufacturer. In brief, approximately 2 ×10^7^ OCI-LY1 or U2932 cells were digested in RIP lysis buffer supplemented with proteinase and RNase inhibitors, cell lysate was divided into input, IP, and IgG groups, followed by overnight incubation at 4 °C with magnetic beads against control immunoglobulin G (5 μg) or NAT10 (ab194297, Abcam, USA) (5 μg). Next, the RNA-protein complexes were isolated by incubating cell lysates with the protein A/G magnetic beads at 4 °C for 1 h. After proteinase K digestion, protein-bound RNAs were extracted by phenol/chloroform/isoamyl alcohol (25:24:1). After that, SLC30A9 mRNA was assessed by qRT-PCR to assess the interaction between NAT10 and SLC30A9. The primers for RIP-qPCR are presented in **Supplementary Table S3**. The protein-bound RNAs were detected by qRT-PCR and assessed by %Input (%Input = 2−[ΔCtIP-(ΔCtinput-log2 Input Dilution Factor)], Input Dilution Factor = (Volume Input/Volume Input + Volume IP + Volume IgG)−1).

**Cell Counting Kit-8 (CCK-8) assay**

Cell Counting Kit-8 (CCK-8) assay (Dojindo, Japan) was used to assess cell proliferation level. In brief, DLBCL cells were seeded in 96-well plates at 1×10^4^ cells per well and stained with 10 μl CCK-8 per well at a certain point in time. The absorbance at 450 nm was detected by Multiskan GO Microplate Reader (Thermo Scientific, Rockford, IL, USA). CompuSyn software was applied to evaluate the effectiveness of drug combinations, representing by the combination index (CI) values. In particular, CI < 1 indicates synergism, CI=1 indicates an additive effect, and CI > 1 indicates antagonism.

**acRIP-qRT-PCR**

The acRIP-qPCR was implemented by Cloud-Seq Biotech Ltd. Co. The RNA is randomly digested into fragments of 100 nucleotides and incubated with protein A/G beads mixed with anti-ac4C antibody. The RNAs were then purified for future research by qRT-PCR. The primers for acRIP-qPCR are presented as follows: acRIP forward primer: GGTGACCATGTGTGTATGCA, acRIP forward primer: CCTAGGCCAACAGGTATGCT.

***In vivo* subcutaneous xenograft models**

Mice were raised under specific pathogen-free conditions, and experiments were conducted following the Institutional Animal Care and Research Advisory Committee of SPH. The bedding material was changed and sterilized at high temperature every other day, the irradiated sterilized feed was fed, and the drinking water was filtered. Female severe combined immunodeficiency (SCID) beige mice aged 4 weeks were received from Beijing Vital River Laboratory Animal Technology Co., Ltd. (Beijing, China). For the *in vivo* study of NAT10, 1×10^7^ OCI-LY1 cells transfected with negative control (Ctrl) , NAT10 knockout (NAT10 KO), empty vector (Vec) , lentivirus-mediated NAT10 overexpression (NAT10 OE) were injected subcutaneously into the flanks of mice in a 200 μl mixture of PBS and Matrigel (356234, Corning, USA). For the efficacy of drug combination studies, xenograft models were established in 4-week-old female SCID beige mice by injecting 1×10^7^ OCI-LY1 cells into the flanks of mice. When tumors reached 100 mm^3^, tumor-bearing mice were randomized into specified groups. The mice received twice-weekly doses of 5 mg/kg remodelin (Selleck, S7641) via intraperitoneal injection, either alone or in combination with 25 mg/kg ibrutinib (Selleck, S2680) via oral gavage. The tumor volume was calculated using V = length × width^2^/2.

**References**

1 Ganapathi, K. A., Brown, L. E., Prakash, S. & Bhargava, P. New developments in non-Hodgkin lymphoid malignancies. *Pathology* **53**, 349-366 (2021). <https://doi.org/10.1016/j.pathol.2021.01.002>

2 Cai, Y. *et al.* Activation of STING by SAMHD1 Deficiency Promotes PANoptosis and Enhances Efficacy of PD-L1 Blockade in Diffuse Large B-cell Lymphoma. *Int J Biol Sci* **19**, 4627-4643 (2023). <https://doi.org/10.7150/ijbs.85236>

3 Chen, X. *et al.* KIAA1429-mediated m6A modification of CHST11 promotes progression of diffuse large B-cell lymphoma by regulating Hippo-YAP pathway. *Cell Mol Biol Lett* **28**, 32 (2023). <https://doi.org/10.1186/s11658-023-00445-w>
