# Supplementary material for: N‐acetyltransferase 10 facilitates tumorigenesis of diffuse large B‐cell lymphoma by regulating AMPK/mTOR signalling through N4‐acetylcytidine modification of SLC30A9
Source: Clin Transl Med. 2024 Jul 3;14(7):e1747. doi: 10.1002/ctm2.1747 (PMC11222071; doi:10.1002/ctm2.1747)
Supplement: Supplementary file 2 — Supporting Information [file CTM2-14-e1747-s001.docx]

**Supplementary Tables**

**Table S1. shRNA sequences for the shNAT10 and shSLC30A9**

| shNAT10#1 | cgCAAAGTTGTGAAGCTATTT |
| --- | --- |
| shNAT10#2 | cgAGCTGGATTTGTTCCTGTT |
| shNAT10#3 | gcAATTGTACACAGTGACTAT |
| shSLC30A9#1 | gcGCTATATTTCTTCGCTAAT |
| shSLC30A9#2 | gcGAGTTGTTACAAGATCATA |
| shSLC30A9#3 | ccTTACTTCTATAACAGGCAA |

**Table S2. The primers for qRT-PCR**

| NAT10 forward | 5’-GGTGGTCTGTCTGGTGGAAG-3’ |
| --- | --- |
| NAT10 reverse | 5’-TCGCTGCTTACGGTGTGAAT-3’ |
| SLC30A9 forward | 5’-TGGGCGAGTTGTTACAAGAT-3’ |
| SLC30A9 reverse | 5’-TGTCGAACTTCAGGATTTCGTTTT-3’ |
| GAPDH forward | 5’-GCACCGTCAAGGCTGAGAAC-3’ |
| GAPDH reverse | 5’- TGGTGAAGACGCCAGTGGA-3’ |

**Table S3. The primers for RIP-qPCR**

| SLC30A9 forward | 5’-TGGGCGAGTTGTTACAAGAT-3’ |
| --- | --- |
| SLC30A9 reverse | 5’-TGTCGAACTTCAGGATTTCGTTTT-3’ |
| GAPDH forward | 5’-GCACCGTCAAGGCTGAGAAC-3’ |
| GAPDH reverse | 5’- TGGTGAAGACGCCAGTGGA-3’ |
